# Ivy leaf (*Hedera helix*) for acute upper respiratory tract infections: an updated systematic review

**DOI:** 10.1007/s00228-021-03090-4

**Published:** 2021-02-01

**Authors:** Elizabeth Sierocinski, Felix Holzinger, Jean-François Chenot

**Affiliations:** 1 Department of General Practice, Institute for Community Medicine, Universitätsmedizin Greifswald, Greifswald, Germany; 2 Department of General Practice, Charité – Universitätsmedizin Berlin, Berlin, Germany

**Keywords:** Acute cough, Bronchitis, Ivy leaf extract, *Hedera helix*

## Abstract

**Purpose:**

Acute cough due to viral upper respiratory tract infections (URTIs) and bronchitis is a common reason for patients to seek medical care. Non-antibiotic over-the-counter cough medications such as ivy leaf extract are frequently used but their efficacy is uncertain. Our purpose was to update our previous systematic review and evaluate the effectiveness and tolerability of ivy leaf in the treatment of acute URTIs in adult and pediatric populations.

**Methods:**

We searched MEDLINE, EMBASE, the Cochrane Library, and clinical trial registries from December 2009 to January 2020. Randomized controlled trials (RCTs), controlled clinical trials (CCTs), and observational studies (OSs) investigating ivy leaf mono- or combination preparations were included. Two independent reviewers assessed records for eligibility and risk of bias and performed data extraction.

**Results:**

Six RCTs, 1 CCT, and 4 OSs were identified. Since the publication of our previous review, the number of RCTs has increased. All studies concluded that ivy leaf extract is an effective and safe option for the treatment of cough due to URTIs and bronchitis. Three RCTs reported a more rapid reduction in cough severity and/or frequency under ivy leaf treatment. The clinical significance of these effects appears to be minimal. No serious adverse effects were reported. The overall quality of reporting was low and the risk of bias was high.

**Conclusions:**

Ivy leaf preparations are safe for use in cough due to acute URTIs and bronchitis. However, effects are minimal at best and of uncertain clinical importance.

**Supplementary Information:**

The online version contains supplementary material available at 10.1007/s00228-021-03090-4.

## Introduction

Acute cough is one of the most common reasons for an individual to seek physician care and to require sick leave from work or school [1, 2]. Viral upper respiratory tract infections (URTIs) and acute bronchitis are the most common cause of acute cough [1] and are hallmarked by general malaise, low or no fever, sore throat, rhinitis, congestion, headache, muscle aches, and cough. Systemic symptoms typically recede after 2–3 days but cough may persist for several weeks [3].

Antibiotics for viral URTIs and bronchitis are ineffective and even harmful due to potential side effects as well as the contribution to the development of bacterial resistance [4]. Despite widespread knowledge of the associated risks, antibiotics are frequently prescribed to patients with URTIs and bronchitis [5]. To combat this issue and to assist physicians in the challenge of alleviating acute cough caused by viral illnesses, a strong evidence base regarding the efficacy and safety of non-antibiotic cough remedies in adults and children is needed.

Ivy leaf (*Hedera helix*) extract preparations are widely used over-the-counter, non-antibiotic cough remedies authorized by the European Medicines Agency [6–8]. Ivy leaf extract contains saponins which are believed to have expectorant properties [9]. In vitro studies of ivy mono-preparations show evidence of potential antispasmodic and bronchodilating activity, anti-inflammatory effects, and antitussive properties [9]. This review is an update of our systematic review published in 2011 which found that evidence for the efficacy of ivy leaf extract in acute cough was inconclusive due to lack of methodologically robust data [10]. The objective of this review was to identify and evaluate new data regarding the effectiveness and tolerability of ivy leaf in the symptomatic treatment of acute bronchitis associated with acute URTIs in children and adults.

## Methods

### Search methods

We conducted a systematic literature search of MEDLINE, EMBASE, and the Cochrane Library from December 2009 until January 2020. Search strategies are available as supplementary material. We hand-searched the bibliographies of retrieved publications and manufacturer websites. Additionally, we searched the World Health Organization International Clinical Trials Registry Platform (WHO ICTR), ClinicalTrials.gov, the European Union Clinical Trials Register (EU CTR), and European Network of Centres for Pharmacoepidemiology and Pharmacovigilance (ENCePP) for ongoing and completed trials and observational studies. We included records in English, German, French, Spanish, and Polish.

### Study selection

Randomized controlled trials (RCTs), controlled clinical trials (CCTs), and non-controlled observational studies (OSs) were included.

### Participants

The target participants were adults and children with upper respiratory tract infections (URTIs) and bronchitis. Studies including other acute diseases such as chronic obstructive pulmonary disease (COPD) and asthma were only included if the majority of subjects had URTIs or bronchitis.

### Interventions

Herbal expectorants in any dosage containing ivy leaf extract either as a single agent or in combination with other herbal agents were targeted.

### Outcomes

We targeted clinical outcomes (e.g., morbidity, health-related quality of life); surrogate values (spirometric parameters); physical findings (auscultation); symptom (cough); and tolerability assessment by physicians or patients.

### Data extraction and management

Two independent reviewers (JFC and ES) screened records for inclusion and extracted data using a predesigned template. Disagreements were resolved by consensus.

### Risk of bias assessment

Two independent reviewers used the Cochrane Risk-of-Bias tools for Randomized Trials (RoB-2) and Non-randomized Studies of Interventions (ROBINS-I) to assess the outcome- and study-level level risk of bias of RCTs and CCTs/OSs, respectively [11, 12]. Disagreements were resolved by consensus. Financial conflicts of interest and publication bias were also assessed [13].

### Data synthesis and subgroup analysis

Included studies were categorized by study design. For controlled studies, the following subgroup comparisons were planned: ivy leaf extract vs. placebo; ivy leaf extract vs. conventional therapy; comparison of different formulations of ivy leaf extract. ROB figures were generated using *robvis* software [14]. All other figures were generated using *drawi.io*.

The review protocol is published on PROSPERO (CRD42019141405).

## Results

### Description of studies

**Fig. 1:**
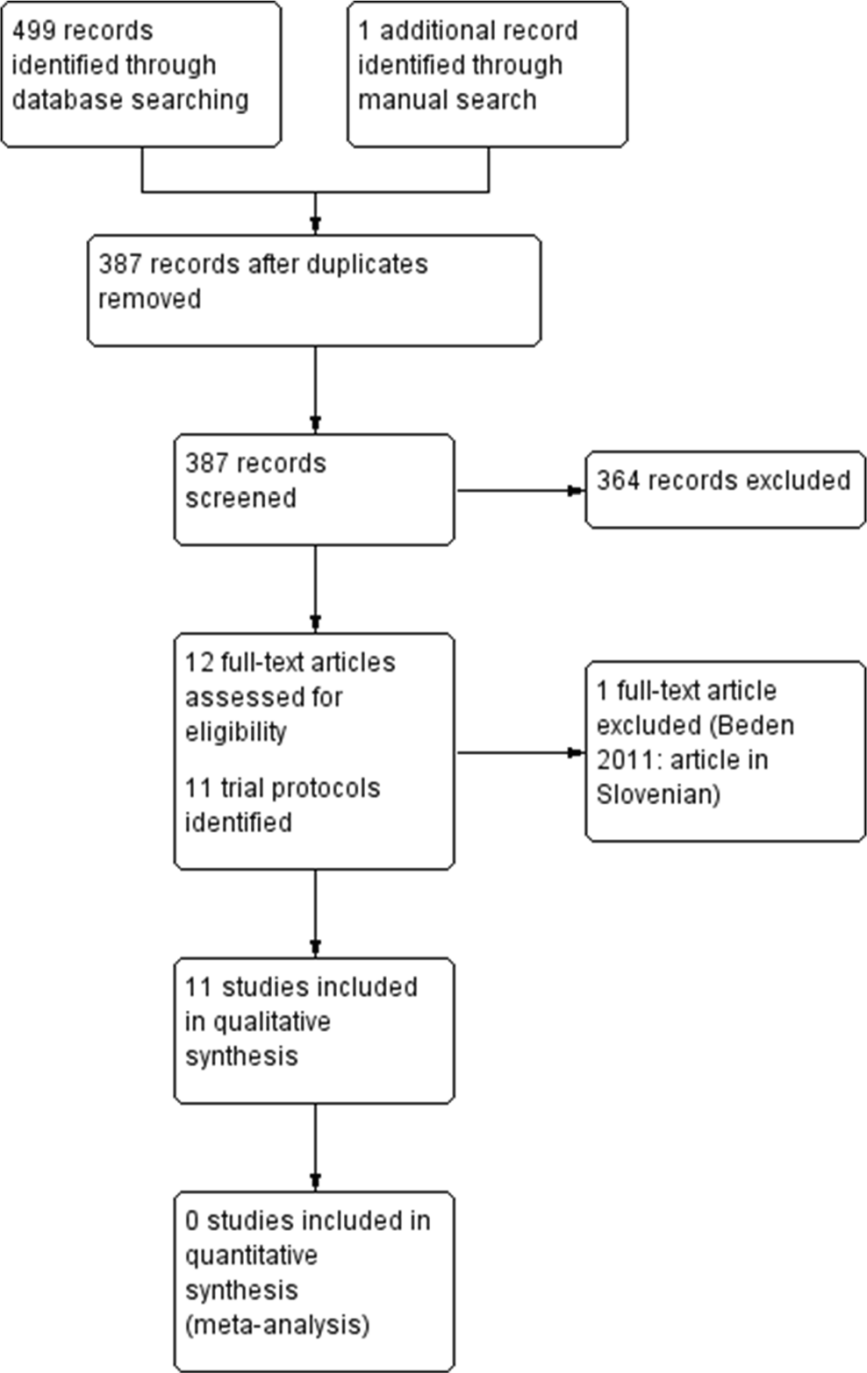
Study selection process

We identified 387 potentially relevant records, including 11 trial protocols (Online Resource 1). Four protocols corresponded to studies included in our review and 7 lacked published results. One full-text article was excluded due to a language barrier [15] and 11 studies were included (Fig. 1) [15].

The studies included 3592 patients (Tables 1 and 2). Two RCTs did not differentiate between acute and chronic cough, although chronic lung diseases such as COPD and asthma were excluded [16, 17]. One RCT included only patients with recurrent acute URTIs (≥ 6/year) [18]. Three OSs included subjects with pneumonia [19] and chronic respiratory diseases [20, 21].

**Table 1:** Characteristics of included studies

Reference	Country	Setting	Protocol pub.	Flow chart	Power calc.	Patients (I/C)a	Inclusion criteria	C/A	Gender (m/f %)	Age range (y)	Intervention group		Control group		Treatment (d)
											Treatment	Daily dose	Treatment	Daily dose	
Randomized controlled trials (RCTs)															
Schaefer 2019	Germany	5 sites: 3 GP, 2 ENT practices	Y	Y	Y	139/70	Acute bronchitis, symptomatic ≤ 2–3 d, BSS > 10, VCD > 2, VAS > 50 mm	0/209	49.3/50.7	18–73	EA 575 (Prospan®) syrup *H. helix* 105 mg DE/5 mL, DER 5-7.5:1, E30%	15 mL	Placebo	n.a.	7
Khan 2018	Pakistan	2 clinics	N	N	N	75/75	Acute and chronic cough, common cold, flu, dry and productive cough	126 (3–15 y)/24 (> 15 y)	53.3/46.6	3 to > 15	“Cough (EMA)” granules *A. officinalis* (marsh-mallow) 1000 mg/10 mL, *S. irio* (London rocket) 100 mg/10 mL, *H. helix* 70 mg/10 mL	1 sachet TID	Placebo	n.a.	7
Ali 2017	Pakistan	Hospitals	N	N	N	110/110	Acute and chronic cough, dry and productive cough	200 (3–15 y)/20 (> 15 y)	48.2/51.8	3 to > 15	“Cofnovex plus (EMA)” syrup *A. officinalis* 1000 mg/10 mL, *S. irio* 100 mg/10 mL, *H. helix* 70 mg/10 mL	NR	Placebo	n.a.	NR
Schaefer 2016	Germany	5 sites: 4 GP, 1 ENT practice	Y	Y	Y	89/92	Acute bronchitis, symptomatic ≤ 2–3 d, BSS > 10, VCD > 2, VAS > 50 mm	0/181	51.4/48.6	18–75	EA 575 (Prospan®) syrup *H. helix* 105 mg DE/5 mL, DER 5-7.5:1, E30%	15 mL	Placebo	n.a.	7
Safina 2014	Russia	1 hospital	N	N	N	28/26	Recurrent acute, mild to moderate respiratory virus infections (≥ 6/y), symptomatic ≤ 2 d	54/0	46.3/53.7	1–6	Bronchipret® syrup *T. vulgaris* 1.5 g LE/10 g, DER 1:2/2.5; *H. helix* 0.15 g/10 g, DER 1:1, E7% Standard care: warm alkaline mineral water, antipyretic (paracetamol), decongestant nose drops, local antibiotic (fusafungin spray) as needed^b^	Age-dependent (y) 1: 2.2 mL 2–6: 3.2 mL	Standard care	n.a.	7–10
Cwientzek 2011	Czech Republic	7 centers	Y	Y	Y	295/295	Acute bronchitis, symptomatic ≤ 48 h, BSS ≥ 5	11% 2–4 y 23% 4-10 y 66% >10 y (max 86 y)	47.0/53.0	2–86	Hedelix® drops *H. helix* 40 mg/5 mL, DER 2.2-2.9:1, E50%	Age-dependent (y) >10: 300 mg 4-10: 200 mg 2-4: 150 mg	Prospan® drops 20 mg DE/mL, DER 5-7.5:1, E30%	Age-dependent (y) >10: 50.4 mg 4-10: 33.6 mg 2-4: 25.2 mg	7 ± 1
Controlled trials (CCTs)															
Khan 2019	Pakistan	1 clinic	N	Y	N	30/30	Acute cough, cold and flu, dry and productive cough	9/51	48.3/51.7	12–60	“Mukalbion” tablets *A. officinalis*, *S. irio*, *H. helix*	2 tablets BID	Poly-herbal marketed brand^c^	2 tablets QID	15
Observational studies (OSs)															
Schön-knecht 2017	Poland	38 sites: GP, Ped, All, Pulm practices	Y	N	N	464	Productive cough of various etiology	464/0	43.0/57.0	2–-12	Hedussin ® syrup *H. helix* 33 mg DE/4 mL, DER 4-8:1, E30%	Age-dependent (y, BID) 2-–5: 2 mL 6-–12: 4 mL	n.a.	n.a.	17 +± 13^d^
Lang 2015	Germany	201 GPs/Peds	N	N	N	1066	Acute bronchitis, viral or bacterial	1066/0	50.4/49.2	6-–12	EA 575 (Prospan®) syrup, drops, effervescent tablets, or lozenges *H. helix* 105 mg DE/5 mL, DER 5-7.5:1, E30%	Various	n.a.	n.a.	6.92 +± 0.05
Schmidt 2012	Germany	6 centers	N	Y	N	268	Acute and chronic bronchitis	268/0	47.3/52.7	0–-12	Hedelix® syrup or cough drops *H. helix* 40 mg/5 mL, DER 2.2-2.9:1, E50%	Age-dependent (y) > 10: 300 mg 4-–10: 200 mg 2-–4: 150 mg 0-–1: 50 mg	n.a.	n.a.	10 (avg.)
Stauss-Grabo 2011	Germany	10 doctors	N	N	N	330	Cough from URTI or chronic respiratory diseases	36 (10-–20 y) / 294 (21-–85 y)	37.6/62.4	11-–85	EA 575 (Prospan®) cough drops *H. helix* 105 mg DE/5 mL, DER 5-7.5:1, E30%	Various: 2 tabs BID, 2 tabs TID, or 1 tablet TID	n.a.	n.a.	8 (med.)

*All* allergologists, *avg.* average, *BID* twice daily, *BSS* Bronchitis Severity Scale, *C/A* children/adults, *d* days, *DE* dry extract, *DER* drug to extract ratio, *E%* ethanol % in extraction solvent, *ENT* ears-nose-throat specialist, *GP* general practitioners or family physicians, *I/C* number in intervention/control group, *LE* liquid extract, *med.* median, *N* no, *n.a.* not applicable, *NR* not reported, *Ped* pediatricians, *Pulm* pulmonologists, *QID* four times daily, *tab(s)* tablet(s), *TID* three times daily, *VAS* visual analog scale, *VCD* verbal cough diary, *y* years, *Y* yes

Values are reported as stated in the respective publications. If information is missing (e.g., composition of herbal preparation), the study in question does not provide details

^a^Number of participants included in analysis

^b^Local antibiotic spray given in case of “acute tonsillopharyngitis”

^c^Combination of: *M. nigra*, liquorice DE, *A. vasica* DE, *O. basilicum* DE, menthol, anisi oil, eucalyptus oil, pine oil, cubeb oil, cinnamon oil

^d^Average follow-up time was 17 ± 13 d (median: 8; range: 5–60); 79.53% had follow-up between 7 and 14 d

**Table 2:**
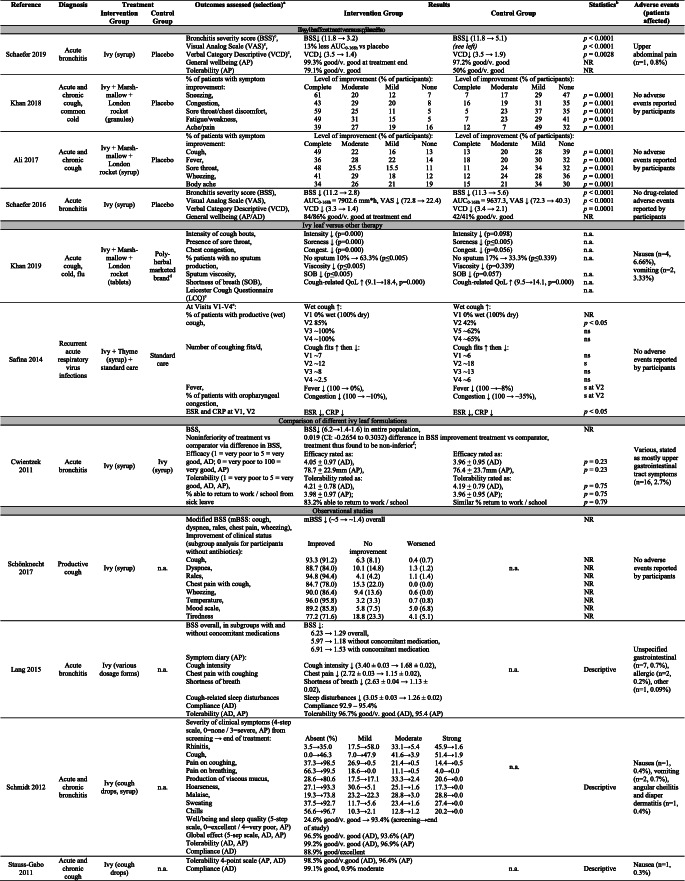
Summary of results of included studies

*d* days, *n.a.* not applicable, *NR* not reported, *ns* not statistically significant, *s* statistically significant, *v*. very, *Abx* antibiotics, *AUC*_*0-168h*_ area under the curve over 7 days, *AD* assessment by doctor, *AP* assessment by patient or caregiver in the case of children, *CI* confidence interval, *QoL* quality of life, ^a^only outcome parameters reported in a manner allowing for comparison between groups are listed (RCT/CCT), for OS: selected relevant outcomes, ^b^if reported: *p* value for intervention compared to control. For OS: *p* value for baseline compared to after treatment, ^c^for detailed context, explanation (where applicable), validation and minimal clinically significant difference of this and other outcome measures, see Online Resource 2, ^d^Combination of: *Morus nigra*, liquorice dry extract, *Adhatoda vasica* dry extract, *Ocimum basilicum* dry extract, menthol, anisi oil, eucalyptus oil, pine oil, cubeb oil, cinnamon oil. ^e^V1 took place on day of study entry, V2 after 3–4 days, V3 after 7–10 days, and V4 after 14–17 days. ^f^The difference in BSS improvement was used to determine noninferiority of treatment vs. comparator (noninferiority margin = − 0.6208); “~” indicates that the value was extrapolated from a figure and no exact value was reported in the study

### Risk of bias

**Fig. 2:**
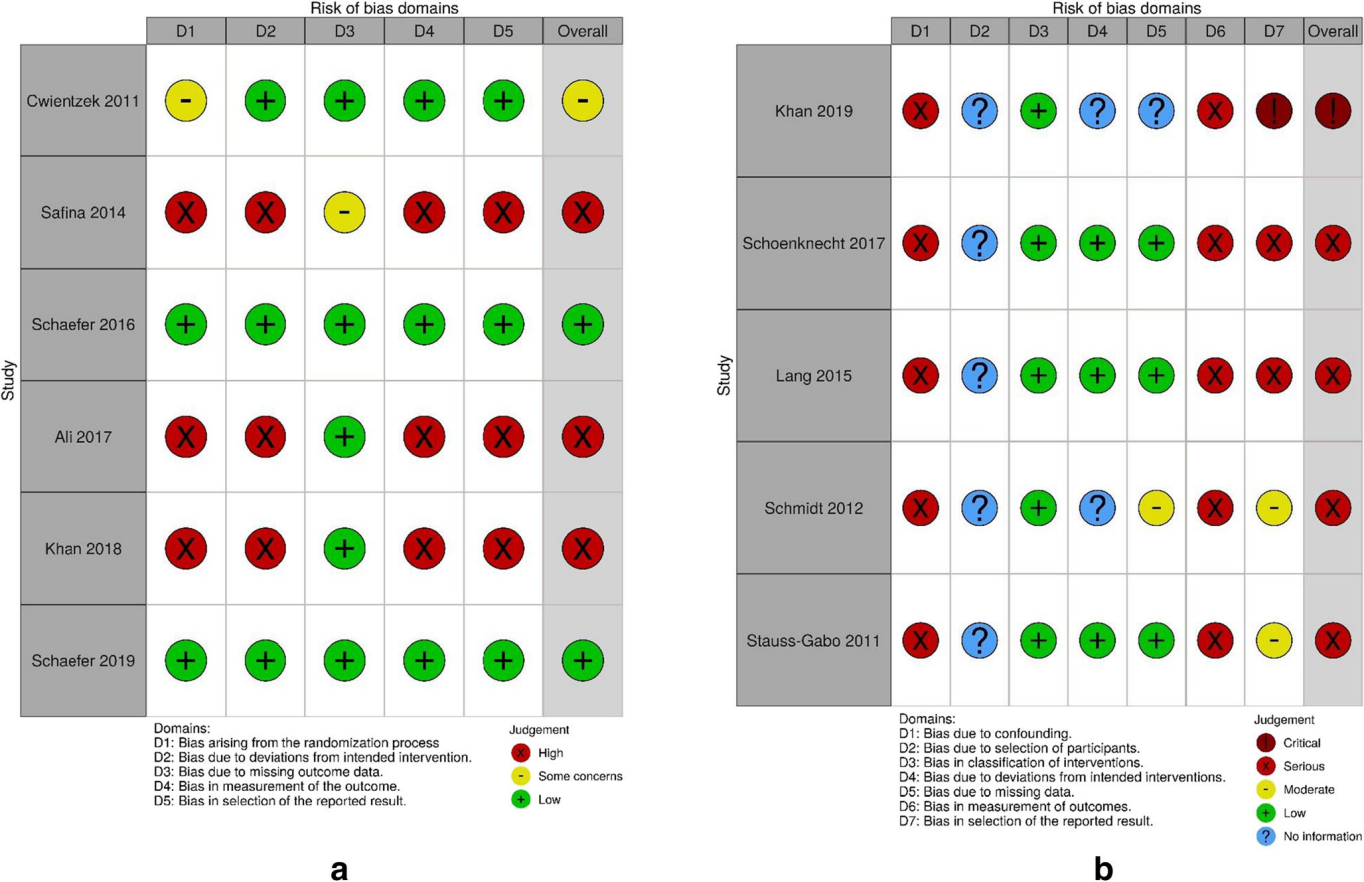
Cochrane risk of bias assessment for **a**) RCTs, based on five domains and ranging from low to high; **b**) non-randomized studies, based on seven domains and ranging from low to critical

Of the RCTs, 2 were found to be at low risk, 3 at high risk, and 1 with some concerns for bias (Fig. 2a). Sources of bias included inadequately described randomization, lack of blinding (2 single-blind [16, 17], 1 open-label [18]), incomplete baseline data, subjective outcome measurements, and selective reporting of results. Of the non-randomized studies, 4 were found to be at serious and 1 at critical risk of bias (Fig. 2b). Sources of bias included uncontrolled confounders, subjective and unblinded measurement of outcomes, and selective reporting of results. Several RCTs and OSs explicitly allowed concomitant medication for the target condition (expectorants [18] and antipyretics [18, 22–24] or antibiotics [18, 19, 25]).

Overall, most studies were found to be at risk of selection bias due to inadequate descriptions of populations screened for eligibility and selection processes.

### Financial conflicts of interest

Six studies declared sponsorship [19, 20, 22–24] or were commissioned by [20] pharmaceutical companies. Five studies did not report funding sources. Of these, 3 were affiliated with the manufacturer of the investigational product [16, 17, 21] and 2 received medications from pharmaceutical companies but did not report affiliations [18, 26].

### Effects of interventions

#### Ivy versus placebo

**Fig. 3:**
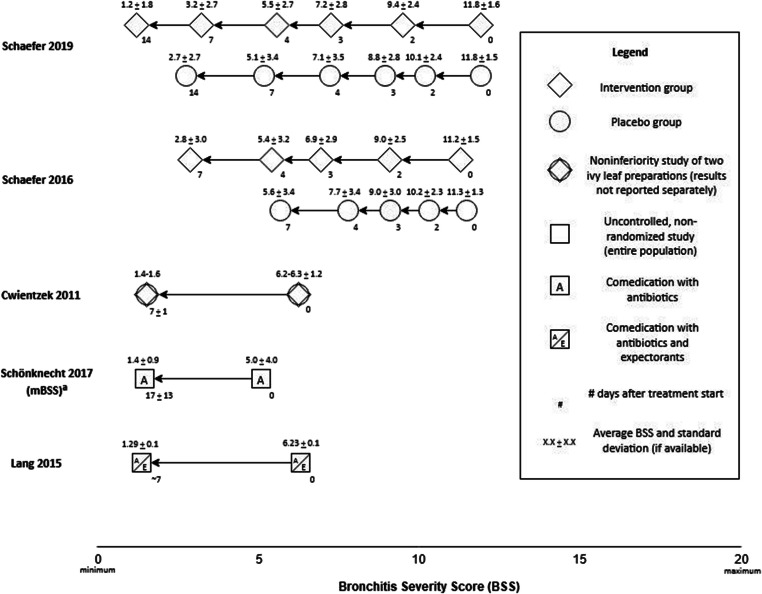
Five heterogeneous studies reporting cough severity via the Bronchitis Severity Scale (BSS) in four cases and a modified BSS (mBSS, wheezing instead of sputum assessed) in one case. Intervention groups are represented by a diamond shape and placebo groups by a circle. A combined circle and diamond shape depicts the BSS for a noninferiority study which combined data for the intervention and comparator groups. The populations of non-randomized studies are represented by squares; text within the squares indicates which comedication was allowed

Two double-blinded RCTs compared an ivy mono-preparation to placebo in 390 adults over 7 days [23, 24] (Table 2). Cough severity was measured by the Bronchitis Severity Scale (BSS) and Visual Analog Scale (VAS) and cough frequency by the Verbal Category Descriptive (VCD) scale (Fig. 3). Statistically significant differences in BSS, VAS, and VCD improvement favoring ivy treatment were reported by treatment day 3.

Two single-blinded RCTs compared an ivy/marsh-mallow/London rocket preparation to placebo in 370 adults and children over 7 days [16, 17]. Physician-measured improvements in cough, congestion, sore throat/chest discomfort, fatigue/weakness, fever, and body ache were reported. A higher percentage of moderate and complete symptom resolution in the intervention group compared to the control was reported.

#### Ivy versus other therapies

One open-label RCT compared an ivy/thyme preparation to standard care, defined as warm alkaline mineral water, an antipyretic (paracetamol), decongestant drops, and a local antibiotic (fusafungin) in 54 children for 7–10 days [18]. Outcomes included the proportion of patients with wet cough and congestion, number of daily coughing fits, fever, and laboratory markers of inflammation. The difference in coughing fits was statistically significant at treatment days 3–4 (approximately 12 vs. 18 fits/day in treatment versus standard care groups, respectively), but not at subsequent follow-up.

The CCT compared an ivy/marsh-mallow/London rocket preparation to a poly-herbal comparator in 60 adults and children over 15 days [26]. Cough intensity, throat soreness, congestion, sputum production and viscosity, and shortness of breath were measured using an unspecified method. Cough-related quality of life (QoL) was measured via the Leicester Cough Questionnaire (LCQ, Online Resource 2). A statistically significant decrease in all symptoms in the investigational group compared to baseline was reported.

#### Different ivy formulations

A double-blinded, randomized noninferiority trial including 590 adults and children compared two ivy mono-preparations for 6–8 days [22]. Outcomes included BSS improvement, physician- and patient-/parent-rated efficacy and tolerability, and percent of patients able to return to school or work. The BSS decreased in the entire study population, with a nonsignificant difference between groups.

#### Observational studies

The four prospective OSs investigated ivy mono-preparations in 2128 patients, 86.2% of which were children [19–21, 25]. Three OSs included concomitant antibiotics and cold medications (e.g., decongestants, nasal sprays) in analyses [19, 21, 25]. Of these, one OS included a subgroup analyses; patients who did not receive antibiotics showed slightly higher percentages of clinical worsening [19].

#### Adverse events and tolerability

All studies recorded data on adverse events, most of which were gastrointestinal [20–22, 24–26]. Two mild unspecified allergic reactions were reported [25] and one isolated skin reaction was possibly related to an ivy mono-preparation [20]. Patient-reported tolerability was reported as good or very good overall [20–22, 24, 25].

#### Heterogeneity of studies

Four studies measured cough severity using the BSS [22–25] and 1 modified the BSS to include wheezing instead of sputum [19] (Fig. 3). The heterogeneity of study designs, inclusion criteria, and treatments precluded meta-analysis.

## Discussion

### Summary of main results

We identified 6 RCTs, 1 CCT, and 4 OSs. Compared to our previous review, the number of RCTs investigating ivy preparations in acute URTIs and bronchitis has increased. All studies concluded that ivy leaf extract is safe. Three RCTs reported a more rapid reduction in cough severity and/or frequency under ivy treatment compared to placebo or standard care. Study heterogeneity precluded quantitative synthesis and meta-analysis. With the exception of two studies, the overall quality of reporting was low and risk of bias was high.

### Effectiveness

Measuring the efficacy of therapies for acute URTIs and bronchitis is challenging as symptoms typically recede after 5–11 days, regardless of intervention [27]. Correspondingly, the clinical condition of participants improved in both treatment and comparison groups. Values for the minimal clinically important difference (MID), or the smallest change perceived by patients as important, are available for two of the tools used to measure cough severity in the studies in our review: 17 mm for the Visual Analog Scale (VAS) and 2 points for the Leicester Cough Questionnaire (LCQ, Online Resource 2) [28]. One RCT reported VAS differences of 11.1 and 17.9 mm between treatment and placebo groups at day 3 and at the end of the treatment period, respectively [23]. Based on the MID, the effect of ivy leaf treatment at 3 days was likely too small to be perceived as important by patients but the difference after 7 days was potentially clinically noticeable. The CCT reported an LCQ difference of 4.3 at the end of the treatment period, indicating a potentially clinically noticeable difference [26].

Half of the RCTs investigated combination preparations which included other active herbal ingredients in addition to ivy leaf extract [16–18]. It is possible that effects described by these studies may be due to the other herbal ingredients or synergy with ivy. The noninferiority trial comparing two different mono-preparations of ivy leaf extract established the equivalency of the test products [22] but did not provide evidence for efficacy. Regarding OSs, conclusions regarding efficacy cannot be drawn due to study design; however, these studies suggest safety and tolerability of ivy preparations.

### Applicability of evidence

Inclusion criteria and population selection varied. Three studies drew participants from specialist (ear-nose-throat, allergology, pulmonology) practices [19, 23, 24], 1 from family medicine and pediatric practices [25], and 7 did not specify source population [16–18, 20–22, 26]. One RCT only included patients with recurrent respiratory tract infections [18]. Specialist referrals often occur in complicated cases or when diagnostic and/or therapeutic options are exhausted in primary care [29], and recurrent infections may indicate more severe underlying disease [30]. This decreases the applicability of these results to our target population of patients with uncomplicated URTIs and bronchitis.

### Completeness of evidence

We identified 11 trial protocols, 7 without published results. Of these, 6 were RCTs completed 2 or more years prior to our search. Results are typically published within 2 years of trial completion and up to 50% of results are never published [31]. We interpret this as evidence of publication bias and postulate that data regarding treatment efficacy is missing from the literature. Given that positive, statistically significant results are more likely to be published than negative or nonsignificant results [32], the unpublished results may describe a lack of efficacy.

### Quality of evidence

Two included studies were at low risk of bias per the Cochrane assessment. This is a minimal improvement to our previous systematic review, in which 1 of 10 included studies was of robust quality per the Jadad scale [10]. All but 1 of the remaining studies were at high or critical risk of bias. The standard of reporting was poor. Half of the RCTs and all OSs measured cough severity subjectively using unblinded outcome assessors. Comedication was expressly allowed in 5 studies [18, 21, 23–25] and not specified in 3 [16, 17, 26], limiting the validity of conclusions*.*

Nine studies were at risk of bias due to financial conflict of interest resulting from manufacturer sponsorship or affiliation. Studies funded by drug companies are 4 times more likely to report favorable outcomes and are at higher risk of publication bias and bias due to inappropriate comparisons [33, 34]. The results of the industry-funded studies included in this review are thus less likely to be generalizable [34].

### Results in context

Four included studies [21, 23–25] also appear in a review of the efficacy of ivy mono-preparation EA 575 in acute and chronic cough [35]. Contrary to our conclusions, this review concludes that EA 575 is efficacious in treating cough. Possible explanations for this difference include the lack of assessment of quality, risk of bias, and clinical significance, as well as bias resulting from financial conflicts of interest [35]. Compared to our 2011 systematic review on this topic [10], the newly published studies are, with few exceptions, of a similarly low methodological quality and continue to show potential bias due to funding by manufacturers.

The majority of adverse events reported by the included studies were of mild to moderate severity and gastrointestinal in nature, corresponding to other publications citing gastrointestinal complaints as the main side effect of ivy preparations [9]. Rare serious adverse events such as anaphylaxis have been reported in the literature [36, 37], but were not reported by the studies in this review.

### Strengths and limitations

Our comprehensive search of major medical databases and supplementary manual search identified studies from multiple countries. Despite manual searching, studies in journals not listed in MEDLINE or EMBASE may have been missed. We had to exclude one study that may have been eligible for inclusion due to a language barrier (Slovenian).

### Authors’ conclusions

#### Implications for practice

Ivy preparations may lead to a marginal reduction in cough symptoms compared to the naturally self-limiting course of URTIs. However, the clinical significance of these effects appears to be minimal. Serious adverse reactions are unlikely.

#### Implications for research

Given the minimal treatment effects reported in the current literature and the natural course of URTIs and bronchitis, it seems unlikely that high-quality, large-scale studies will establish clinically important effects.

## Supplementary Information

ESM 1 (DOCX 18 kb)

## Data Availability

Complete search strategies are available in Online Resource 3.
