# Supplementary material for: Ivy leaf (Hedera helix) for acute upper respiratory tract infections: an updated systematic review
Source: Eur J Clin Pharmacol. 2021 Feb 1;77(8):1113–22. doi: 10.1007/s00228-021-03090-4 (PMC8275562; doi:10.1007/s00228-021-03090-4)
Supplement: Supplementary file 1 — (DOCX 18 kb) [file 228_2021_3090_MOESM1_ESM.docx]

Online Resource 1. Study protocols identified and applicable studies.

| **Registry** | **Year published** | **ID** | **Title** | **Design** | **Country** | **Sponsor** | **Published end date** | **Published results** |
| --- | --- | --- | --- | --- | --- | --- | --- | --- |
| EU PAS | 2017 | EUPAS21094 | Assessment of the efficacy of the Hedussin® medicinal product use in the treatment of productive (wet) cough | OS | Poland | Europharma Rachtan | 2017-10-15 | Schönknecht 2017 |
| ClinicalTrials.gov | 2017 | NCT02981147 | Ivy, Thyme and Cisti Extract (Phytus) Efficacy in Acute Cough (PEACe) | Open-label trial | Pakistan | PharmEvo Pvt Ltd | 2018-03-30 | None |
| EudraCT | 2016 | 2016-002426-37 | Randomized, placebo-controlled, double-blind, multi-center trial to evaluate the efficacy and safety of 2 Prospan® posologies (2x 7.5 mL/day and 3x 5 mL/day) in the treatment of acute bronchitis | RCT | Germany | Engelhard Arzneimittel GmbH | 2017-09-23 | Schaefer 2019 |
| ClinicalTrials.gov | 2015 | NCT02396706 | RCT to Evaluate the Efficacy and Safety of Ivy Leaves Cough Liquid vs. Placebo in the Treatment of Acute Cough | RCT | Germany | Engelhard Arzneimittel GmbH | June 2015 | None |
| ClinicalTrials.gov | 2014 | NCT02045550 | Tolerance and Effect of a Prophylactical Treatment with Ivy Leaves Dry Extract in Recurrent Wheezy Bronchitis | RCT | Germany | Technische Universität Dresden | August 2015 | None |
| EudraCT | 2014 | 2014-003590-41 | Randomized, controlled, double-blind, multi-center trial to evaluate the efficacy and safety of a liquid containing ivy leaves dry extract vs. placebo in the treatment of acute cough | RCT | Germany | Engelhard Arzneimittel GmbH | 2015-08-25 | Schaefer 2016 |
| EudraCT | 2010 | 2009-016778-34 | Evaluation de la non-infériorité de l’efficacité d’Helicidine® versus Sirop à base de dextromethorphane sur la toux sèche des patients pris en charge en médecine générale. | RCT | France | Therabel Lucien Pharma | unknown | None |
| Clinicaltrials.gov | 2009 | NCT01151202 | Therapeutic Confirmatory Clinical Trial to Evaluate the Safety and Efficacy of "AG NPP709 Syrup" | RCT | South Korea | Ahn-Gook Pharmaceuticals Co.,Ltd | June 2010 | None |
| ClinicalTrials.gov | 2008 | NCT01127048 | Prospan® Hustenzäpfchen - Investigation on Efficacy and Tolerability in Children | RCT | Germany | Engelhard Arzneimittel GmbH | August 2013 | None |
| EudraCT | 2008 | 2008-000473-38 | Prospan® Hustenzäpfchen – Investigation on efficacy and tolerability in children: A double-blind, placebo controlled, randomised, multi-centre, comparative study with parallel groups for the symptomatic treatment of acute bronchitis accompanied by coughing in children | RCT | Germany | Engelhard Arzneimittel GmbH | 2011-12-28 | None |
| EudraCT | 2007 | 2007-003272-19 | Acute bronchitis therapy with ivy leaves extracts in a two-arm study: a double-blind, ransomised study vs. comparator | RCT | Czech Republic | Krewel Meuselbach GmbH | 2009-04-03 | Cwientzek 2011 |

EU PAS: European Union electronic Register of Post-Authorisation Studies, searchable via the European Network of Centres for Pharmacoepidemiology and Pharmacovigilance (ENEPP)
EudraCT: European Union Drug Regulating Authorities Clinical Trials Database, searchable via the European Union Clinical Trials Register (EU CTR).

Online Resource 2. Tools and questionnaires used to measure outcomes in included studies

| Tool | Papers using tool | Scale | Validation for acute cough | Minimally important difference (MID)* |
| --- | --- | --- | --- | --- |
| Bronchitis Severity Scale (BSS) | Schaefer 2019  Schaefer 2016 | Cough, sputum, rales on auscultation, chest pain with coughing, and dyspnea assessed by clinician according to a 5-point Likert scale (0 = absent, 4 = very severe). Points allocated to each symptom/sign add up to a total score of 0-20. | Validated for measuring severity of acute bronchitis (Lehrl 2014)  Validated for pediatric populations (Lehrl 2018) | n.a. |
| Visual Analog Scale (VAS) | Schaefer 2019  Schaefer 2016 | Patients draw an X to indicate cough severity on a printed 100-mm scale ranging from no cough (0 mm) to worst cough (100 mm) (Spinou 2014). | Validated for measurement of cough (Spinou 2014) | The smallest change in VAS that subjects perceived to be important was 17 mm from baseline (Lee 2013). |
| Verbal Category Descriptive (VCD) | Schaefer 2019  Schaefer 2016 | Cough and the level to which it interferes with daily activities assessed on a 6-point Likert scale (0 = no cough, 5 = cannot perform most usual activities due to coughing). | n.a. | n.a. |
| Leicester Cough Questionnaire (LCQ) | Khan 2019 | 19 questions assessing physical, psychological, social domains as affected by cough for a score range 3 (not impaired) to 21 (quality of life most impaired) (Lee 2013/ Birring 2003) | Validated for acute cough (Yousaf 2011) | 2 points are the smallest clinically relevant change noticed by patients (Lee 2013). |
| "Considering all the ways this treatment has affected you since you started in the clinical trial, how well are you doing?" | Schaefer 2019  Schaefer 2016 | 5-choice Likert scale: very poor, poor, fair, well, very well. | n.a. | n.a. |
| The Clinical Global Impression – Severity scale (CGI-S) | Lang 2015 | 7-point scale that requires the clinician to rate the severity of the patient's illness at the time of assessment, relative to the clinician's past experience with patients who have the same diagnosis. Possible ratings are: 1. Normal, not at all ill 2. Borderline ill 3. Mildly ill 4. Moderately ill 5. Markedly ill 6. Severely ill 7. Among the most extremely ill patients | n.a. | n.a. |

n.a. = not applicable, *minimal amount of change in parameters considered important by patients.

**References**

Birring, S. S., et al. (2003). "Development of a symptom specific health status measure for patients with chronic cough: Leicester Cough Questionnaire (LCQ)." Thorax 58(4): 339-343.

Kardos, P., et al. (2014). "The BSS - A Valid Clinical Instrument to Measure the Severity of Acute Bronchitis." Journal of Lung, Pulmonary & Respiratory Research 1(3): 72-80.

Lee, K. K., et al. (2013). "A longitudinal assessment of acute cough." Am J Respir Crit Care Med 187(9): 991-997.

Lehrl, S., et al. (2018). "Validation of a Clinical Instrument for Measuring the Severity of Acute Bronchitis in Children - The BSS-ped." Open Respir Med J 12: 50-66.

Spinou, A. and S. S. Birring (2014). "An update on measurement and monitoring of cough: what are the important study endpoints?" J Thorac Dis 6(Suppl 7): S728-734.

Yousaf, N., et al. (2011). "The assessment of quality of life in acute cough with the Leicester Cough Questionnaire (LCQ-acute)." Cough 7(1): 4.

Online Resource 3. Search Strategies.

**PubMed.**

*Search 1.*

"Hedera"[Mesh]

Filters activated: Publication date from 2009/12/20, Humans.

Translates to detailed search:

"Hedera"[Mesh] AND (("2009/12/20"[PDAT] : "3000/12/31"[PDAT]) AND "humans"[MeSH Terms])

*Search 2.*

(hedera OR ivy) AND (respiratory tract diseases OR respirat* OR lung OR asthma OR copd OR cough OR bronchitis OR bronchial OR obstructive)

Filters activated: Publication date from 2009/12/20, Humans.

**Cochrane Library.**

*Search 1.*

MeSH descriptor: [Hedera] explode all trees

Filter applied: Date added to database after 12/20/2009

*Search 2.*

(hedera OR ivy) AND (respiratory tract diseases OR respirat* OR lung OR asthma OR copd OR cough OR bronchitis OR bronchial OR obstructive)

Filter applied: Date added to database after 12/20/2009

**Embase.**

*Search 1 syntax.*

1 exp ivy/

2 limit 1 to (human and yr="2009 -Current")

*Search 2 syntax.*

1. hedera.mp

2. ivy.mp.

3. respiratory tract diseases.mp. or respiratory tract disease/

4. respirat*.mp.

5. lung.mp.

6. asthma.mp. or asthma/

7. copd.mp. or chronic obstructive lung disease/

8. bronchitis.mp. or bronchitis/

9. cough.mp. or exp coughing/

10. bronchial.mp.

11. obstructive.mp.

12. 1 or 2

13. 3 or 4 or 5 or 6 or 7 or 8 or 9 or 10 or 11

14. 12 and 13

15. limit 14 to (human and yr="2009 -Current")
